# Rhizobacteria-Mediated Activation of the Fe Deficiency Response in Arabidopsis Roots: Impact on Fe Status and Signaling

**DOI:** 10.3389/fpls.2019.00909

**Published:** 2019-07-12

**Authors:** Eline H. Verbon, Pauline L. Trapet, Sophie Kruijs, Coline Temple-Boyer-Dury, T. Gerrit Rouwenhorst, Corné M. J. Pieterse

**Affiliations:** ^1^Plant-Microbe Interactions, Department of Biology, Science4Life, Utrecht University, Utrecht, Netherlands; ^2^Ecology and Biodiversity, Department of Biology, Science4Life, Utrecht University, Utrecht, Netherlands

**Keywords:** plant growth-promoting rhizobacteria, Fe deficiency response, *Arabidopsis thaliana*, *Pseudomonas simiae* WCS417, shoot-to-root signaling

## Abstract

The beneficial root-colonizing rhizobacterium *Pseudomonas simiae* WCS417 stimulates plant growth and induces systemic resistance against a broad spectrum of plant diseases. In *Arabidopsis thaliana* (Arabidopsis), the root transcriptional response to WCS417 shows significant overlap with the root response to iron (Fe) starvation, including activation of the marker genes *MYB72* and *IRT1*. Here, we investigated how colonization of Arabidopsis roots by WCS417 impacts Fe homeostasis in roots and shoots. Under Fe-sufficient conditions, root colonization by WCS417 induced a transient Fe deficiency response in the root and elevated both the total amount of Fe in the shoot and the shoot fresh weight. When plants were grown under Fe-starvation conditions, WCS417 still promoted plant growth, but did not increase the total amount of Fe, resulting in chlorosis. Thus, increased Fe uptake in response to WCS417 is essential to maintain Fe homeostasis in the more rapidly growing plant. As the WCS417-induced Fe deficiency response is known to require a shoot-derived signal, we tested whether the Fe deficiency response is activated in response to an increased Fe demand in the more rapidly growing shoot. Exogenous application of Fe to the leaves to reduce a potential shoot Fe shortage did not prevent WCS417-mediated induction of the Fe deficiency response in the roots. Moreover, the leaf Fe status-dependent shoot-to-root signaling mutant *opt3-2*, which is impaired in the phloem-specific Fe transporter OPT3, still up-regulated the Fe deficiency response genes *MYB72* and *IRT1* in response to WCS417. Collectively, our results suggest that the WCS417-induced Fe deficiency response in the root is controlled by a shoot-to-root signaling system that functions independently of both leaf Fe status and OPT3.

## Introduction

The composition of the microbial community in the soil surrounding plant roots is clearly different from that in soil further away from the roots. This phenomenon, known as the rhizosphere effect, is associated with the secretion of carbon sources by plant roots. These carbon sources can serve as nutrients for soil microbes (Berendsen et al., 2012; Lundberg et al., 2012) or as selective agents that shape the composition of the root microbiome (Bakker et al., 2018; Stringlis et al., 2018b). In return, plant growth-promoting rhizobacteria (PGPR) within the root microbiome can improve plant growth and health (Lugtenberg and Kamilova, 2009; Pieterse et al., 2014; Berendsen et al., 2018). The PGPR *Pseudomonas simiae* WCS417 (hereafter: WCS417) is among the most well-studied PGPRs. When WCS417 colonizes the root of the model plant *Arabidopsis thaliana* (hereafter: Arabidopsis), it stimulates plant growth and induces systemic resistance (ISR) against a broad variety of pathogens (Zamioudis et al., 2013; Pieterse et al., 2014). WCS417-ISR is not associated with immediate upregulation of defense responses in the leaves. Instead, the increased resistance is associated with a more rapid and stronger activation of defense responses upon pathogen attack, a cost-effective form of induced resistance known as defense priming (Martinez-Medina et al., 2016). In the roots, WCS417 actively suppresses local defense responses triggered by its microbe-associated molecular patterns (MAMPs) (Stringlis et al., 2018a), possibly to facilitate colonization and promotion of plant growth. Apart from downregulating defense responses, WCS417 mediates activation of the root-specific transcription factor gene *MYB72* and its downstream target *β-GLUCOSIDASE42* (*BGLU42*). These genes are essential for the onset of WCS417-ISR in Arabidopsis, as the mutants *myb72* and *bglu42* do not mount systemic immunity upon colonization of the roots by WCS417 (Van der Ent et al., 2008; Zamioudis et al., 2014). *MYB72* is also required for ISR triggered by other beneficial microbes, including the beneficial fungus *Trichoderma asperellum* T-34 (Segarra et al., 2009; Martínez-Medina et al., 2017).

*MYB72* and *BGLU42* are not only involved in WCS417-ISR, they are also part of the iron (Fe) deficiency response that is initiated in plant roots under conditions of Fe starvation (Palmer et al., 2013; Zamioudis et al., 2014; Verbon et al., 2017). Interestingly, 20% of all genes induced by WCS417 in Arabidopsis roots are also induced under Fe-limited conditions (Zamioudis et al., 2015), providing evidence for a mechanistic link between the Fe deficiency response and ISR (Zamioudis et al., 2014; Verbon et al., 2017). Like WCS417, the beneficial ISR-inducing fungi *T. asperellum* T-34 and *Trichoderma harzianum* T-78 also induce the Fe deficiency response in Arabidopsis and tomato (Martínez-Medina et al., 2017), supporting the notion activation of the Fe deficiency response by beneficial microbes is a wide-spread phenomenon (Romera et al., 2019).

Plant Fe deficiency responses are elaborate molecular mechanisms that increase Fe uptake when plants experience Fe shortage (Römheld, 1987; Walker and Connolly, 2008; Ivanov et al., 2012; Kobayashi and Nishizawa, 2012). This is essential for plant growth and health as Fe is required as an enzyme cofactor in many essential processes, such as respiration, DNA synthesis and photosynthesis (Briat et al., 1995). Even though Fe is abundantly present in the Earth’s crust, its bioavailability is limited because Fe is mainly present as ferric oxide, which is poorly soluble at neutral and high pH. Arabidopsis, like other non-grass plants, utilizes the root-specific Strategy I Fe deficiency response to safeguard sufficient Fe uptake under Fe starvation conditions (Römheld, 1987). In Strategy I, Fe mobilization is realized by members of plasma membrane-localized H^+^-ATPases, which secrete protons to acidify the rhizosphere and thereby enhance the solubility of ferric Fe (Fe^3+^) in the soil (Colangelo and Guerinot, 2004). The basic helix-loop-helix (bHLH) transcription factor FIT (FER-LIKE IRON DEFICIENCY TRANSCRIPTION FACTOR) functions as a central regulator of Strategy I, as it regulates the expression of a number of critical Fe uptake genes, including *FRO2*, which encodes the enzyme FERRIC REDUCTION OXIDASE2 that reduces soluble Fe^3+^ to ferrous Fe (Fe^2+^), and *IRT1*, which encodes the high-affinity IRON-REGULATED TRANSPORTER1 that transports Fe^2+^ into the plant root (Colangelo and Guerinot, 2004).

In addition to the core Strategy I genes, FIT regulates *MYB72* gene expression (Colangelo and Guerinot, 2004; Zamioudis et al., 2015). The root-specific transcription factor MYB72 and its paralogue MYB10 are required for plant survival when Fe availability is limited (Palmer et al., 2013). MYB72 regulates the biosynthesis and secretion of a subclass of Fe-mobilizing phenolic compounds called coumarins (Zamioudis et al., 2014; Stringlis et al., 2018b, 2019). Downstream of MYB72, activity of the glucoside hydrolase BGLU42, which converts glycosylated coumarins into their aglycone counterparts, is required for secretion of coumarins into the rhizosphere (Zamioudis et al., 2014; Stringlis et al., 2018b). Among the metabolites whose biosynthesis and secretion are dependent on MYB72 and BGLU42, the coumarin scopoletin is the most abundant (Stringlis et al., 2018b). Coumarins are synthesized in the phenylpropanoid pathway via FERULOYL-COA 6′-HYDROXYLASE1 (F6′H1) (Rodriguez-Celma et al., 2013; Schmid et al., 2014) and are secreted into the rhizosphere by the Fe deficiency-regulated ABC transporter PLEIOTROPIC DRUG RESISTANCE9 (PDR9) (Rodriguez-Celma et al., 2013; Fourcroy et al., 2014). In addition to *MYB72* and *BGLU42*, several other genes with roles in the biosynthesis and secretion of Fe-mobilizing coumarins are induced in Arabidopsis roots upon colonization by WCS417, even when plants are grown under Fe-sufficient conditions (Zamioudis et al., 2014). These include *F6*′*H1*, *MYB10*, *SCOPOLETIN 8-HYDROXYLASE* (*S8H*) (Rajniak et al., 2018; Tsai et al., 2018), *CYP82C4* (Rajniak et al., 2018), and *PDR9*. Upon release in the rhizosphere, coumarins can chelate and mobilize Fe^3+^ and make it available for reduction and uptake by the roots, therewith improving Fe nutrition of the plant (Schmid et al., 2014; Fourcroy et al., 2016; Tsai and Schmidt, 2017). Interestingly, some coumarins, such as scopoletin, possess a selective antimicrobial activity that can help the plant to shape its microbiome in the rhizosphere in favor of coumarin biosynthesis-activating PGPR, such as WCS417, and improve plant health (Stringlis et al., 2018b, 2019). Possibly, WCS417 hijacks the Fe deficiency response to trigger production and secretion of the selective antimicrobial coumarins to improve its own niche establishment in the rhizosphere.

In Arabidopsis roots, the WCS417-induced Fe deficiency response, including the activation of *MYB72* and *IRT1*, is under the control of a shoot-to-root signaling system (Zamioudis et al., 2015). This is also the case for the regulation of the canonical Fe deficiency response (Grusak and Pezeshgi, 1996). After Fe is taken up from the soil into root epidermal cells, it moves symplastically toward the vasculature from where it is transported to the shoot (Hindt and Guerinot, 2012). The plasma membrane transporter OPT3, which loads Fe from the xylem into the phloem, regulates leaf Fe status shoot-to-root signaling to maintain Fe homeostasis and prevent Fe overload (Zhai et al., 2014; Khan et al., 2018). Misregulation of leaf Fe shoot-to-root signaling in mutant *opt3-2* plants results in a constitutively active Fe deficiency response in the roots, even under Fe-sufficient conditions (Stacey et al., 2008). Maintaining Fe homeostasis is important to plant health (Aznar et al., 2015; Verbon et al., 2017) and Fe overload should be avoided because it results in oxidative stress (Connolly and Guerinot, 2002). To prevent Fe concentrations building to toxic levels, Fe storage in the plant is tightly controlled. Ferritins (FERs) are important players in this process (Ravet et al., 2009). The expression of *FER* genes is typically upregulated when Fe content in the plant increases (Briat et al., 1999). FERs can subsequently store up to 4500 Fe atoms in their cavity, thereby preventing free Fe from inducing oxidative stress (Briat et al., 1999; Ravet et al., 2009).

In recent years, several studies demonstrated that beneficial soil-borne microbes can improve Fe nutrition of plants, and that this is linked to their ability to trigger ISR (Romera et al., 2019). However, the biological mechanisms driving this microbial effect on Fe nutrition are not fully understood. In this study, we investigated how WCS417 affects Fe homeostasis in Arabidopsis. Moreover, we investigated the role of leaf Fe status in the shoot-to-root signaling-dependent activation of the Fe deficiency response by WCS417. Our results show that increased Fe uptake in response to colonization of the roots by WCS417 is essential to support WCS417-induced plant growth promotion. In addition, we show that the WCS417-induced activation of the Fe deficiency response is independent of Fe status-regulated shoot-to-root signaling.

## Materials and Methods

### Plant Material and Growth Conditions

Seedlings of *A. thaliana* accession Col-0 and mutant *opt3-2* (Stacey et al., 2008) were grown on a piece of nylon mesh (Nitex Cat 03-100/44, Sefar, Heiden, Switzerland) on standard Fe-sufficient growth medium consisting of modified Hoagland medium (Hoagland and Arnon, 1938) containing 5 mM KNO_3_, 2 mM MgSO_4_, 2 mM Ca(NO_3_)_2_, 2.5 mM KH_2_PO_4_, 70 μM H_3_BO_3_, 14 μM MnCl_2_, 1 mM ZnSO_4_, 0.5 mM CuSO_4_, 10 μM NaCl, 0.2 μM Na_2_MoO_4_, 0.05% 2-ethanesulfonic acid (MES; Duchefa Biochemie, Haarlem, Netherlands), 50 μM FeNaEDTA, 1% sucrose, and 1% plant agar (Duchefa Biochemie). The pH of the medium was set to 5.7. Fe-deficient medium was prepared by omitting FeNaEDTA from the standard medium.

Typically, Arabidopsis seeds were sown at low density on standard Fe-sufficient growth medium (a single row of twenty seeds per square Petri dish of 120 × 120 mm). Seeds were sterilized by a 3-h exposure to the gas formed upon mixing 100 ml bleach with 3.2 ml hydrochloric acid fuming (37%) (Van Wees et al., 2013). After sowing, plates were sealed with a double layer of Parafilm and stratified in the dark at 4°C for 48 h. Plates were then placed vertically in a growth chamber under short-day conditions (14-h night, 10-h day; 21°C; 100 μmol m^–2^ s^–1^).

### Bacterial and Fe Deficiency Treatments

On the day of PGPR inoculation, after either 5 or 12 days of growth on standard Fe-sufficient medium in the short-day growth chamber, plants were transferred to new standard Fe-sufficient or to Fe-deficient plates by moving the nylon mesh with the plants on top to the fresh plates. One day before transfer of the plants to fresh medium and inoculation, the PGPR strain *P. simiae* WCS417 (Berendsen et al., 2015) was streaked from a frozen glycerol stock onto King’s B medium agar plates (King et al., 1954) and incubated overnight in the dark at 28°C. The next day, a bacterial suspension was prepared as described previously (Zamioudis et al., 2015). In brief, bacteria were collected from the overnight plates, washed twice in 10 mM MgSO_4_, and then suspended in 10 mM MgSO_4_ to a final density of OD_600_ = 0.01, or 0.001 when specified. Plants were inoculated by applying 10 μl of the bacterial suspension halfway down each root. The remaining plants were similarly treated with 10 μl of 10 mM MgSO_4_ (mock) or remained untreated (Control). After treatment, plates were closed with a double layer of Parafilm and returned to the short-day growth chamber. Plant material was harvested 1–7 days later.

### Application of Fe Supplement to the Shoot

For shoot supplementation with different concentrations of Fe, 12-day-old Col-0 plants were transferred to new standard Fe-sufficient plates. Subsequently, 0.2 μl of MilliQ (mock) or 0.2 μl of a 5 μM, 50 μM, 500 μM, or 5 mM FeNaEDTA solution was added to two leaves of each plant, immediately after inoculation of the roots with WCS417. After 2 and 7 days, roots and shoots were harvested for gene expression analysis and shoot fresh weight (FW) measurement. Material for gene expression analysis was snap frozen in liquid nitrogen and stored at −80°C until RNA isolation.

### Segmented Plate Assays

Segmented plates (square Petri dishes 120 × 120 mm) were prepared by removing a 5-mm strip of medium from an Fe-deficient plate, rendering two physically separated pieces of Fe-deficient medium, as described previously (Giehl et al., 2012). The strip was removed at 20 mm from the outer side of the Petri dish. Fe concentrations in the small top part of the medium were amended to a calculated final concentration of 40 μM or 200 μM FeNaEDTA by adding 42 or 205 μl, respectively, of 8 mM FeNaEDTA. The Fe concentration in the large bottom part was amended to a final calculated concentration of 40 μM FeNaEDTA by adding 208 μl of 8 mM FeNaEDTA. Plates were left horizontally at room temperature (RT) overnight to allow the Fe to diffuse into the medium. Five-day-old seedlings were transferred on their nylon mesh to the segmented plates with the shoot part touching the small top part of the medium and the root part touching the large bottom part of the plate. Bacterial treatments in this system were performed by applying 10 μl of a bacterial suspension (WCS417 at OD_600_ = 0.01) halfway down each root system, immediately after transfer of the plants to the segmented plates. Plants were decapitated just prior to treatment with WCS417 through removal of the shoot by cutting directly below the hypocotyl as described previously (Zamioudis et al., 2015). Roots were harvested 2 days after transfer of the plants to the segmented plate system. Plant material was stored at −80°C until RNA isolation.

### Quantitative Reverse Transcription-Polymerase Chain Reaction (qRT-PCR)

RNA was isolated from frozen roots and shoots (Oñate-Sánchez and Vicente-Carbajosa, 2008) and prepared for qRT-PCR as described previously (Caarls et al., 2017). In short, cDNA was synthesized from DNase-treated total RNA samples using an oligo-dT primer. PCR reactions were performed using SYBR^®^ green to monitor the synthesis of double-stranded DNA. Gene expression was analyzed using the comparative Ct method (Schmittgen and Livak, 2008). First, gene expression was normalized to the expression level of the reference gene *PP2AA3* (At1g13320) by subtracting the cycle threshold (*C*_t_) of *PP2AA3* from the Ct of the gene of interest in the same sample, generating Δ*C*_t_ values. ΔΔ*C*_t_ values were calculated by taking the average *C*_t_ of the control samples and subtracting this value from the Δ*C*_t_ values of the treated samples. Statistical analyses were performed on the ΔΔ*C*_t_ values. Relative gene expression (fold change in gene expression relative to control), calculated as 2^–ΔΔ^*^Ct^*, was plotted.

### Plant Growth and Chlorophyll Measurements

For growth measurements, shoots were cut from the roots just below the hypocotyl. Shoots from all the plants grown on a single plate were counted and pooled to obtain one biological replicate. The shoots were gently dried with tissue paper to remove any adhering moisture and weighed. Average shoot FW was calculated by dividing the total shoot FW by the number of plants on the plate. Chlorophyll was measured from the same samples as described previously (Hiscox and Israelstam, 1979). In brief, leaf tissue from the Arabidopsis seedlings from a single plate were placed in a vial containing 3 ml dimethylsulfoxide (DMSO) per 100 mg of shoot FW and incubated for 45 min at 65°C. After cooling to RT, chlorophyll (*a* + *b*) extracts were transferred to a cuvette, and spectrophotometer readings were performed using a DU–64 spectrophotometer (Beckman Coulter, Brea, United States) at a wavelength of 652 nm. Chlorophyll concentrations were calculated as described by Hiscox and Israelstam (1979).

### Fe Measurement

To produce large quantities of plant material for Fe content measurements, the plant growth and PGPR inoculation protocol described above was slightly adjusted. Large batches of seeds were liquid sterilized in 60% bleach (v:v) for 10 min, followed by eight washes with MilliQ water (Dinneny et al., 2008). Seeds were sown at high density in a dense 10-cm row of three seeds thick on a square Petri dish of 120 × 120 mm. Plants grown at high density were inoculated by dividing 125 μl of a bacterial suspension at OD_600_ = 0.01 over the root systems of each row of plants. For Fe measurements, the collective shoot tissue from at least three high-density sown Arabidopsis plates were pooled to obtain one biological replicate. Plants were prepared for Fe content analyses as described previously (Trapet et al., 2016). In brief, shoot material was gently dried with tissue paper and transferred into 50-ml conical tubes with 30 ml of MilliQ water. After 10 min of shaking at RT, plant tissue was washed twice in rinsing solution (5 mM EDTA, 1 mM KCl, 5 mM Na_2_S_2_O_4_, 0.5 mM CaSO_4_⋅2H_2_O, pH 6) at RT for 10 min on a shaker. After a final 10-min wash with MilliQ, the samples were gently dried with tissue paper and transferred into Pyrex tubes. Fe residues had been removed from these tubes by a 3-h rinse in 0.1 M hydrochloric acid, followed by a 1-h rinse in 5 mM EDTA and a final wash in MilliQ. The samples were dried completely by placing them in a 65°C incubator for 2 days. Subsequently, tissues were ground with a glass stick, after which the dry weight was measured. For mineralization, up to 10 mg of dry weight per sample was solubilized in 300 μl 65% nitric acid (Merck, Kenilworth, United States) and incubated at 120°C until all liquid was evaporated. The remaining material was mineralized a second time with 100 μl nitric acid and 200 μl hydrogen peroxide. This second mineralization step was repeated until all the material was mineralized. The mineralized material was dissolved in 200 μl nitric acid and 100 μl hydrogen peroxide. The final volume was subsequently adjusted to 10 ml by adding 9.7 ml of MilliQ. The Fe concentration of the samples was analyzed with an inductively coupled plasma atomic emission spectrometer (Thermo Scientific, Waltham, MA, United States), as described previously. The following settings were used: radio frequency power 1150 W, auxiliary gas flow 0.5 l min^–1^, pump rate 50 rpm, nebulizer gas flow 0.5 l min^–1^, coolant gas flow 12 l min^–1^, optics temperature 38°C, camera temperature 45°C.

## Results

### Characteristics of PGPR-Induced Fe Deficiency Response

**FIGURE 1:**
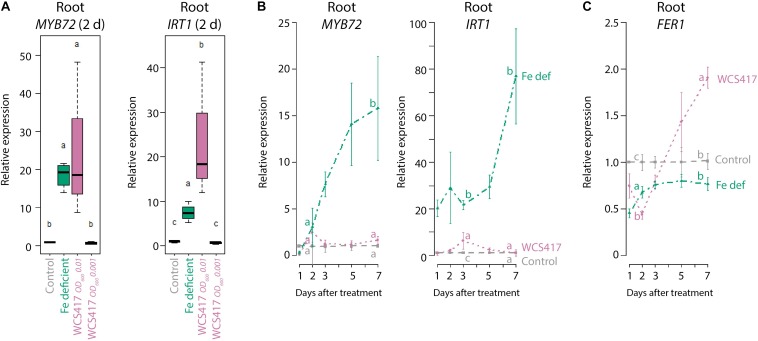
ISR and Fe deficiency marker gene expression in roots in response to Fe starvation or WCS417. **(A)** qRT-qPCR analysis of *MYB72* and *IRT1* gene expression in roots of 7-day-old Col-0 plants at 2 days after transfer to Fe-deficient medium, or inoculation of the roots with a WCS417 suspension at OD_600_ = 0.01 or 0.001. **(B,C)** qRT-PCR analysis of *MYB72*, *IRT1*, and *FER1* gene expression in roots of Col-0 plants at different time points after inoculation of 12-day-old roots with WCS417 at OD_600_ = 0.01, or transfer to Fe-deficient medium (Fe def). Gene expression levels were normalized to that of the constitutively expressed reference gene *PP2AA3* (At1g13320). Plotted are fold changes in gene expression levels relative to that of the average of the control treatment. Error bars represent standard errors of the mean. Per timepoint, different letters indicate statistically significant differences between treatments (one-way ANOVA followed by Tukey’s test; *P <* 0.05; *n* = 4–5).

The *Pseudomonas* spp. PGPR strain WCS417 is able to trigger ISR and promote plant growth in different plant species (Berendsen et al., 2015). In our standard *in vitro* plate system on Fe-sufficient medium, colonization of the roots of Arabidopsis Col-0 by WCS417 increases shoot FW by ∼threefold (Wintermans et al., 2016). Even though the plants grew on Fe-sufficient medium, colonization of the roots by WCS417 (OD_600_ = 0.01) was associated with an increase in the expression of the Fe deficiency response marker genes *MYB72* and *IRT1* (Figure 1A). Interestingly, 2 days after treatment, the increased *MYB72* and *IRT1* mRNA levels in WCS417 OD_600_ 0.01-treated roots growing on Fe-sufficient medium were in the same range (*MYB72*) or even higher (*IRT1*) than in roots growing on Fe-deficient medium (Figure 1A). When monitored over an extended period of 7 days, the expression profiles of *MYB72* and *IRT1* in WCS417-treated roots displayed a transient twofold to fivefold increase, which peaked at day 2 or 3 after inoculation (Figure 1B, pink versus gray lines), while in roots of Fe-starved plants *MYB72* and *IRT1* mRNA levels continued to increase to high levels at 7 days after treatment (Figure 1B, green versus gray lines). Previously, it was shown that a threshold of ISR-stimulating PGPR is required on the roots for the activation of ISR (Raaijmakers et al., 1995). To test if the same holds true for the activation of the marker genes *MYB72* and *IRT1*, we tested the effect of a 10-fold lower bacterial density of WCS417. Application of WCS417 to the root system at a density of OD_600_ 0.001 failed to induce expression of the Fe-deficiency marker genes (Figure 1A), suggesting that the activation of these genes requires a threshold level of bacteria on the root system. Together, these results indicate that PGPR induce the Fe deficiency response genes *MYB72* and *IRT1* even when sufficient Fe is available in the growth medium, but only when they are present at sufficient numbers on the root system. In addition, the PGPR-mediated Fe deficiency response on Fe-sufficient medium is mild and transient compared to the response induced by Fe limitation. Possibly, the WCS417-mediated activation of the Fe deficiency response on Fe-sufficient medium rapidly results in increased Fe uptake, which in turn down-regulates the activated Fe deficiency response.

### Fe Status in PGPR-Colonized Plants

To investigate whether the WCS417-induced Fe deficiency response affects Fe status in the roots, we tested the expression of the Fe storage protein gene *FER1*. *FER1* expression reflects metabolic Fe needs and serves as a robust marker for the cellular Fe status (Gaymard et al., 1996), with *FER1* expression typically high under conditions of Fe excess and low under Fe-deficient conditions (Petit et al., 2001; Arnaud et al., 2006). In Fe-starved plants, *FER1* mRNA levels remained low during the course of the experiment (Figure 1C, green versus gray line). In contrast, in WCS417-treated roots of plants growing on Fe-sufficient medium, *FER1* mRNA levels dropped during the first 2 days, but then increased to levels that were significantly higher than those in control plants growing on Fe-sufficient medium (Figure 1C, pink versus gray line), suggesting that Fe uptake increased in the roots in response to WCS417 colonization.

**FIGURE 2:**
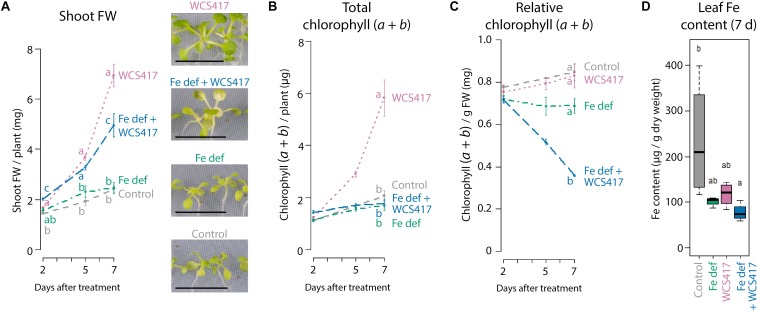
Effect of WCS417 on plant growth and Fe status under Fe-sufficient and Fe-deficient conditions. Shoot FW **(A)**, total and relative chlorophyll (*a* + *b*) content **(B,C)**, and Fe content **(D)** in shoots of control and WCS417-treated Col-0 plants grown on Fe-sufficient medium (gray and purple) or Fe-deficient medium (Fe def; green and blue) at 2, 5, and 7 days after the indicated treatments. Fe deficiency was applied by transferring 12-day-old plants from standard Fe-sufficient medium to Fe-deficient medium. WCS417 inoculation was performed on roots of 12-day-old plants using a bacterial suspension at OD_600_ = 0.01. Photographs are representative pictures from the experiment quantified in A. *MYB72*, *IRT1*, and *FER1* gene expression data corresponding to the shoot FW and chlorophyll and Fe content data are shown in Figure 1. Error bars represent standard errors of the mean. Per timepoint, different letters indicate statistically significant differences between treatments (one-way ANOVA followed by Tukey’s test; *P* < 0.05; *n* = 3–6). Scale bars in panel **(A)** represent 1 cm.

To test whether WCS417 colonization results in enhanced plant Fe uptake, we monitored the effect of WCS417 on chlorophyll and Fe content in the leaves of Col-0 plants growing on Fe-sufficient or Fe-deficient medium. Figure 2A shows that WCS417 significantly increased shoot FW on both Fe-sufficient and Fe-deficient medium, although the increase in shoot FW was more pronounced on Fe-sufficient medium (Figure 2A, pink versus blue line). Interestingly, total chlorophyll content, which is an indicator for Fe nutrition in the leaves (Briat et al., 2015), followed the WCS417-mediated increase in shoot FW when plants were grown on Fe-sufficient medium (Figure 2B, pink versus gray line). However, on Fe-deficient medium, total chlorophyll content per WCS417-treated plant remained constant over time (Figure 2B, blue versus green line), even though plant FW increased in WCS417-treated plants (Figure 2A). As a result, the relative chlorophyll content per gram of shoot FW was significantly lower in WCS417-treated plants growing on Fe-deficient medium (Figure 2C), as was reflected by a more chlorotic appearance of the shoots (Figure 2A). As expected, measurement of the Fe content per gram of dry weight shows the same trend as the chlorophyll content. While Fe content per g of shoot dry weight did not significantly differ between WCS417-treated plants and their respective controls, the Fe content of Fe-starved plants tended to be lower (Figure 2D). For Fe measurements (Figure 2D), the protocol for plant cultivation and inoculation was slightly different from that for the analyses of gene expression, shoot FW, and chlorophyll content (see “Materials and Methods” section). Notwithstanding, in both systems the plants responded similarly to Fe starvation and WCS417 treatment as evidenced by the similar expression patterns of *MYB72*, *IRT1*, and *FER1* (Supplementary Figure S1 and Figure 1). Together, these data suggest that under conditions of sufficient Fe availability, WCS417-enhanced Fe uptake ensures the maintenance of Fe homeostasis in the more rapidly growing plant.

### Shoot-to-Root Fe Signaling in PGPR-Treated Plants

**FIGURE 3:**
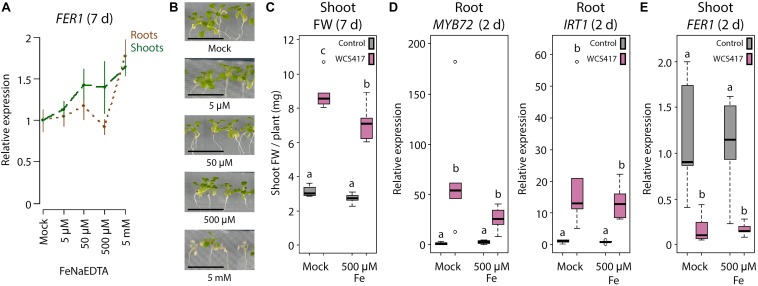
Effect of exogenous Fe supply to shoots on the WCS417-induced Fe deficiency response in roots. **(A)** qRT-PCR analysis of *FER1* gene expression in roots and shoots of 19-day-old Col-0 plants **(B)**, 7 days after exogenous application of indicated FeNaEDTA concentrations to the shoot. **(C)** Shoot FW of WCS417-inoculated and non-inoculated plants, 7 days after shoot Fe supply. **(D,E)** qRT-PCR analysis of *MYB72*, *IRT1*, or *FER1* gene expression in roots or shoots, 2 days after shoot Fe supply. Shoots of 12-day-old Col-0 plants growing on Fe-sufficient medium were supplemented with Fe by applying 0.2 μl of a solution with indicated concentrations of FeNaEDTA onto two leaves per plant. Root inoculations with WCS417 (OD_600_ = 0.01) were performed just prior to supply of Fe to the shoots. Gene expression levels were normalized to that of the constitutively expressed reference gene *PP2AA3* (At1g13320). Plotted are fold changes in gene expression levels relative to the average of non-inoculated, mock-treated plants. Error bars represent standard errors of the mean. Per timepoint, different letters indicate statistically significant differences between treatments (one-way ANOVA followed by Tukey’s test; *P <* 0.05; *n =* 5-6). Scale bars in panel **(B)** represent 1 cm.

Fe uptake by the roots is under the control of leaf Fe status-dependent shoot-derived signals to maintain Fe homeostasis and prevent Fe overload (Grusak and Pezeshgi, 1996; Grillet et al., 2018). Previously, we demonstrated that the WCS417-induced Fe deficiency response in the roots is also controlled by shoot-to-root signaling (Zamioudis et al., 2015). As the Fe deficiency response is induced by WCS417 even when plants are grown under Fe-sufficient conditions (Figure 1) (Zamioudis et al., 2015), we hypothesized that this response is activated due to an enhanced Fe demand in the faster-growing shoots. To test this hypothesis, we studied whether exogenous Fe supply to the leaves prevents WCS417-mediated induction of the Fe deficiency response in the roots. A concentration range of 5 μM–5 mM of exogenously supplied FeNaEDTA gradually increased *FER1* mRNA levels in the shoots of sterile-grown plants at 7 days after treatment, suggesting that Fe content increased in the Fe-supplemented shoots (Figure 3A). The concentration of 500 μM was the highest concentration that could be supplied to leaves without causing visible symptoms of phytotoxicity (Figure 3B), so this concentration was chosen for further experiments. Colonization of the roots by WCS417 promoted growth in Col-0 plants irrespective of whether the leaves were supplemented with 500 μM FeNaEDTA or not (Figure 3C). Application of the Fe supplement to the leaves had no effect on the WCS417-induced levels of *MYB72* and *IRT1* in the roots (Figure 3D), suggesting that leaf Fe shoot-to-root signaling does not affect WCS417-mediated induction of the Fe deficiency response in the roots. The WCS417-mediated growth promotion observed in Figure 3C was associated with a decrease in *FER1* mRNA levels in the control shoots at 2 days after inoculation of the roots with WCS417 (Figure 3E), suggesting that WCS417-treated plants were in demand for Fe (Figure 3D). A similar drop in *FER1* mRNA levels was observed in WCS417-treated plants of which the shoots were supplemented with Fe. Hence, a single application of 500 μM FeNaEDTA to the leaves at the onset of root colonization by WCS417 was not sufficient to compensate for the increased Fe demand in the leaves during the early stages of WCS417-mediated plant growth promotion.

**FIGURE 4:**
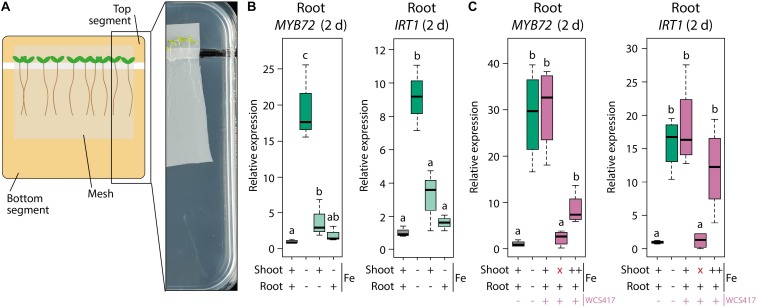
Effect of differential Fe supply to shoots and roots in a segmented plate assay on WCS417-induced Fe deficiency response in roots. **(A)** Schematic representation and picture of the segmented plate assay in which Col-0 plants on nylon mesh are positioned with their shoots on the top segment and with their root systems on the bottom segment of the plate. **(B,C)** qRT-PCR analysis of *MYB72* or *IRT1* gene expression in roots, 2 days after transfer of 5-day-old Col-0 plants onto segmented plates in which the top and bottom segments were supplemented with FeNaEDTA to reach calculated final concentrations of either 0 μM (–), 40 μM (+) or 200 μM (++). Root inoculations with WCS417 (OD_600_ = 0.01) were performed immediately after transfer of the plants to the segmented plates. Decapitation of the shoot (indicated by a red “x”) was performed by cutting at the shoot-root junction just prior to application of WCS417 to the roots. Gene expression levels were normalized to that of the constitutively expressed reference gene *PP2AA3* (At1g13320). Plotted are fold changes in gene expression levels relative to that of non-inoculated plants growing with both shoots and roots on Fe-sufficient medium. Error bars represent standard errors of the mean. Different letters indicate statistically significant differences between treatments (one-way ANOVA followed by Tukey’s test; *P <* 0.05; *n =* 3–4).

In order to supply a more continuous source of exogenous Fe to the leaves we made use of a segmented plate setup in which roots and shoots could be exposed to different Fe concentrations (Figure 4A). When both roots and shoots of sterile-grown plants were cultivated for 2 days on Fe-deficient medium, *MYB72* and *IRT1* were induced in the roots, as expected (Figure 4B). When the shoots were placed on Fe-sufficient medium and the roots on Fe-deficient medium, the levels of *MYB72* and *IRT1* gene expression were significantly reduced (Figure 4B). Hence, in this experimental setup, leaf Fe status-mediated shoot-to-root signaling repressed the Fe deficiency response in the roots when the shoots were not in demand for Fe. Vice versa, when roots were placed on Fe-sufficient medium and shoots on Fe-deficient medium, *MYB72* and *IRT1* were not activated, likely because the roots had ample Fe to take up from the medium to supply the shoots with sufficient Fe (Figure 4B). Next, we tested the effect of WCS417 in this experimental set up. Again, plants of which both shoots and roots were grown on Fe-deficient segments showed increased expression of the Fe deficiency response genes *MYB72* and *IRT1* (Figure 4C). In response to WCS417 root colonization, plants of which both shoots and roots were grown on Fe-sufficient segments showed a similarly induced expression level of *MYB72* and *IRT1* (Figure 4C). Decapitation of the shoot from the root just prior to root inoculation with WCS417 completely prevented activation of *MYB72* and *IRT1* (Figure 4C, red X), confirming previous findings that a shoot-derived signal is required for the onset of the Fe deficiency response in the roots by WCS417 (Zamioudis et al., 2015). To test whether induction of the Fe deficiency response in roots by WCS417 is caused by an increased Fe demand in the shoot, we placed the shoots on medium amended with five times more Fe (++) than the standard Fe-sufficient plates (+). We reasoned that the increased Fe concentration in the top segment would compensate for the potentially enhanced Fe demand in the shoots of WCS417-treated plants. In this setup, the roots were placed on standard Fe-sufficient medium. Figure 4C shows that when the shoots were exposed to a higher concentration of Fe in the medium, WCS417-induced expression of *MYB72* in the roots was reduced, but not statistically significantly. For *IRT1* gene expression, the effect of the enhanced Fe supply via the leaves was even weaker and also not statistically significant. Together, these results point to a scenario in which it is unlikely that an increased Fe demand in the shoots as a result of WCS417-mediated growth promotion is responsible for the shoot-to-root signaling that leads to the activation of the Fe deficiency response in WCS417-colonized roots.

### PGPR-Mediated Shoot-to-Root Fe Signaling Is Independent of OPT3

**FIGURE 5:**
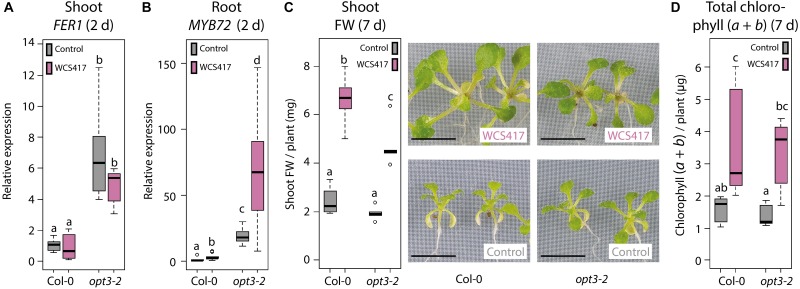
Induction of the Fe deficiency response by WCS417 in leaf Fe status shoot-to-root signaling mutant *opt3-2*. qRT-PCR analysis of *FER1* gene expression in shoots **(A)** and *MYB72* gene expression in roots **(B)** of 14-day-old Col-0 and *opt3-2* plants growing on Fe-sufficient medium, 2 days after inoculation of the roots with WCS417 (OD_600_ = 0.01). **(C)** Shoot FW and representative pictures of Col-0 and *opt3-2* plants, 7 days after inoculation of the roots with WCS417. **(D)** Total chlorophyll (*a* + *b*) content of Col-0 and *opt3-2* plants, 7 days after inoculation with WCS417. Gene expression levels were normalized to that of the constitutively expressed reference gene *PP2AA3* (At1g13320). Plotted are fold changes in gene expression levels relative to the average of non-inoculated Col-0 plants. Error bars represent standard errors of the mean. Different letters indicate statistically significant differences between treatments (one-way ANOVA followed by Tukey’s test; *P <* 0.05; *n =* 5–10). Scale bars in panel **(C)** represent 0.5 cm.

The above-described results suggest that the WCS417-induced Fe deficiency response in the roots does not rely on the canonical Fe status-mediated shoot-to-root signaling system that communicates whether activation of the Fe deficiency response is required. To find further proof for this conclusion, we made use of the knockdown mutant *opt3-2*, which is impaired in leaf Fe status shoot-to-root signaling (Mendoza-Cózatl et al., 2014; Zhai et al., 2014). Misregulation of shoot-to-root Fe signaling in *opt3-2* results in a constitutively active Fe deficiency response in the roots under Fe-sufficient conditions, resulting in high Fe levels in the shoot (Stacey et al., 2008). This Fe overload is sensed in the shoot, but is not communicated to the root to suppress the Fe deficiency response (Khan et al., 2018). We reasoned that if the Fe deficiency response is activated by WCS417 because of an Fe shortage in the shoot, it should not be activated by WCS417 in the *opt3-2* mutant, as this mutant has an Fe overload in the shoot. To test this, we inoculated the roots of Col-0 and *opt3-2* plants growing on Fe-sufficient medium with WCS417 or a mock solution. The expression level of the Fe storage gene *FER1* was significantly higher in the control shoots of *opt3-2* plants than in those of Col-0 (Figure 5A), which is indicative of the previously reported enhanced Fe levels in *opt3-2* leaves (Stacey et al., 2008). In addition, basal *MYB72* mRNA levels were 19-fold higher in control *opt3-2* roots than in control Col-0 roots (Figure 5B), confirming that *opt3-2* roots constitutively express the Fe deficiency response (Khan et al., 2018). WCS417 promoted growth in both Col-0 and *opt3-2*, resulting in a typical two to threefold increase in shoot FW (Figure 5C) and an increase in the total amount of chlorophyll per plant (Figure 5D). In addition, inoculation of Col-0 roots with WCS417 resulted in a typical threefold increase in *MYB72* expression. In *opt3-2* mutants, *MYB72* transcript levels were also increased by threefold in response to WCS417, from 19- to 53-fold relative to mock-treated Col-0 roots. These results show again that the WCS417-induced Fe deficiency response in roots is not controlled by the canonical Fe status-mediated shoot-to-root signaling pathway but by a shoot-dependent signaling system that functions independently of the Fe status and OPT3.

## Discussion

PGPR in the root microbiome extend the functional repertoire of plants by enhancing nutrient uptake, improving root system architecture, and stimulating the plant immune system (Berendsen et al., 2012). Over the past few years, evidence has accumulated that plants evolved adaptive strategies to attract beneficial root-associated microbiota to optimize both nutrient acquisition and immunity (Hiruma et al., 2016; Castrillo et al., 2017; Verbon et al., 2017; Bakker et al., 2018; Stringlis et al., 2018b). One of the nutrients whose uptake is affected by PGPR is Fe. Fe is among the essential mineral nutrients required by plants, but its bioavailability in the soil is often limited (Palmer et al., 2013; Tsai and Schmidt, 2017). This explains why plants evolved elaborate Fe deficiency responses to increase iron uptake in Fe limiting conditions (Römheld, 1987). Upon interaction with the model PGPR WCS417, Arabidopsis roots activate this Fe deficiency response, even when the interaction takes place in Fe-sufficient conditions (Zamioudis et al., 2014). Activation of the response results in increased production and excretion of MYB72-dependent Fe-mobilizing coumarins (Stringlis et al., 2018b). As some coumarins have selective antimicrobial activity, the PGPR-induced secretion of these coumarins is thought to help the PGPR to establish in their niche on the root. In addition to activating the Fe deficiency response, PGPR can promote plant growth and trigger ISR. Activation of both the Fe deficiency response and ISR is a common phenomenon in interactions between dicot plants and beneficial microbes (Romera et al., 2019). However, little is known about the mechanism underlying the induction of the Fe deficiency response by PGPR, or about its effect on Fe homeostasis and signaling in the plant. In this study, we show that WCS417 activates the Fe deficiency response only when present in sufficient numbers on the roots. In comparison to conditions of Fe starvation, the WCS417-elicited Fe deficiency response is mild and transient (Figure 1). We further show that the induced Fe deficiency response is not the cause of WCS417-mediated plant growth promotion, as WCS417 still promoted plant growth under Fe-limiting conditions. Nevertheless, WCS417-induced Fe uptake is essential for the plant to maintain Fe homeostasis during the WCS417-enhanced plant growth (Figures 1, 2). Finally, we demonstrate that the WCS417-induced Fe deficiency response in the roots is regulated by a so far unidentified shoot-to-root signaling system that is independent of leaf Fe status and OPT3.

### Elicitation of the Fe Deficiency Response by WCS417 Is Not Caused by Fe Depletion in the Root Environment

Plant root colonization by growth-promoting microbes induces molecular and morphological changes in the roots that resemble those induced under Fe starvation (Zamioudis et al., 2013, 2014; Verbon and Liberman, 2016; Martínez-Medina et al., 2017; Verbon et al., 2017; Romera et al., 2019). Many root microbiota have the capacity to produce Fe-chelating siderophores that ensure microbial uptake of Fe when Fe is scarce (Berendsen et al., 2015; Stringlis et al., 2018c). Interestingly, siderophores have also been shown to play a role in the onset of ISR (Duijff et al., 1993; Meziane et al., 2005; De Vleesschauwer et al., 2008). This raises the question whether PGPR-mediated activation of the Fe deficiency response and ISR are caused by a microbially inflicted Fe depletion in the root environment. However, the expression patterns of the Fe deficiency response marker genes *MYB72* and *IRT1* in Arabidopsis roots in response to root colonization by PGPR versus Fe starvation are different. The PGPR elicited a mild, transient expression pattern, whereas Fe starvation triggered an Fe deficiency response that was much stronger and increased gradually over time (Figure 1). These results support the notion that the PGPR-induced Fe deficiency response is not activated because of a physical depletion of Fe from the root environment but is elicited by a so far unidentified mechanism that functions independently of Fe availability in around the root. This current view is supported by the fact that siderophore mutants of WCS417 are not impaired in their capacity to activate the Fe deficiency response in Arabidopsis roots and that volatiles produced by ISR-inducing microbes can remotely activate the Fe deficiency response (Zamioudis et al., 2015; Martínez-Medina et al., 2017).

### The WCS417-Induced Fe Deficiency Response in the Root Is Independent of Leaf Fe Status

Previously, we showed that a shoot-derived signal is required for the activation of the Fe deficiency response by PGPR in Arabidopsis roots (Zamioudis et al., 2015). As WCS417-mediated activation of the Fe deficiency response supported Fe nutrition in the faster growing plant (Figure 2), we reasoned that the Fe deficiency response might be activated in response to an increased Fe demand in the faster growing shoots. This resembles the previously described leaf Fe status-dependent shoot-to-root signaling system that maintains Fe homeostasis and prevents Fe overload via the regulatory activity of the leaf Fe transporter OPT3 (Zhai et al., 2014; Khan et al., 2018). However, while exogenous Fe supply to the leaves suppressed Fe starvation-mediated activation of the Fe deficiency response in the roots, it did not prevent WCS417-mediated activation of the response (Figures 3, 4). Moreover, the leaf Fe status shoot-to-root signaling mutant *opt3-2*, which builds an Fe overload in its shoots (Zhai et al., 2014; Khan et al., 2018), was still responsive to WCS417-mediated activation of the Fe deficiency response in the roots (Figure 5). We therefore conclude that the Fe acquisition response triggered by PGPR such as WCS417 is under the control of a shoot-to-root signaling system that functions independently from OPT3 and leaf Fe status. Recently, the IRON MAN (IMA) peptide family was discovered as novel phloem-mobile shoot-to-root signals that control Fe uptake in the roots under conditions of Fe starvation (Grillet et al., 2018). Interestingly, *IMA1* is also induced upon colonization of the roots by WCS417 (Zamioudis et al., 2014) and could therefore play a role in the shoot-to-root signaling required for the activation of the Fe deficiency response. However, the role of IMA peptides in PGPR-mediated activation of the Fe acquisition response remains to be resolved.

An alternative scenario for the activation of the Fe deficiency response by WCS417 involves the phytohormone auxin. Auxin is required for the morphological and molecular root responses that are typically observed in roots in response to WCS417 and Fe starvation (Chen et al., 2010; Zamioudis et al., 2013; Stringlis et al., 2018a). While WCS417 does not produce auxin itself, it stimulates an auxin response in Arabidopsis roots (Zamioudis et al., 2013). Possibly as a result, 44% of the WCS417-induced transcriptional changes in Arabidopsis roots are also induced by auxin, including the induction of *MYB72* and *IRT1* (Stringlis et al., 2018a). The stimulation of an auxin response by WCS417 might explain how WCS417 activates both the Fe deficiency response and the observed morphological changes in root system architecture. As the auxin response in the root is also under control of a shoot-to-root signaling system (Ljung et al., 2005), the involvement of auxin signaling could be an explanation for the shoot dependency of the WCS417-mediated activation of the Fe deficiency response. To test this hypothesis, further investigation into the interaction between the signaling pathways induced in response to PGPR, Fe starvation and auxin is required.

### The Fine Balance Between Harmful and Beneficial

Many biological control agents, such as WCS417, promote plant growth, improve plant nutrition, and/or prime the plant immune system in controlled experimental settings (Lugtenberg and Kamilova, 2009; Pieterse et al., 2014; Liu et al., 2017; Ab Rahman et al., 2018). However, biological control agents often show inconsistent performance in the field. In this study, it becomes clear that a switch from beneficial to harmful effects of PGPR can be induced by the environmental conditions in which the interaction takes place. Colonization of the roots by WCS417 promoted plant growth irrespective of whether plants were grown on Fe-sufficient or Fe-deficient medium (Figure 2). On Fe-sufficient medium, the WCS417-induced Fe acquisition response allowed to plant to maintain Fe homeostasis while growing more rapidly. However, under Fe limiting conditions, the faster growing WCS417-stimulated plants had a significantly lower chlorophyll content and became chlorotic. Hence, under specific environmental conditions, when not all prerequisites for the potentially beneficial microbial functions are met, biological control agents can become detrimental for plant performance. This highlights the importance of basic research on understanding the biological mechanisms by which beneficial microbes promote plant growth and control diseases, as it will provide important information that may facilitate the successful application of novel biofertilizers and biopesticides in the field.

## Data Availability

All datasets generated for this study are included in the manuscript and/or the Supplementary Files.

## Author Contributions

EV, PT, and CP initiated the project. EV, PT, SK, and CT-B-D performed the experiments and analyzed the data. TR performed the Fe measurements. EV, PT, and CP wrote the manuscript.

## Conflict of Interest Statement

The authors declare that the research was conducted in the absence of any commercial or financial relationships that could be construed as a potential conflict of interest.
