# Supplementary material for: Rhizobacteria-Mediated Activation of the Fe Deficiency Response in Arabidopsis Roots: Impact on Fe Status and Signaling
Source: Front Plant Sci. 2019 Jul 12;10:909. doi: 10.3389/fpls.2019.00909 (PMC6639660; doi:10.3389/fpls.2019.00909)
Supplement: Supplementary file 1 [file Data_Sheet_1.PDF]

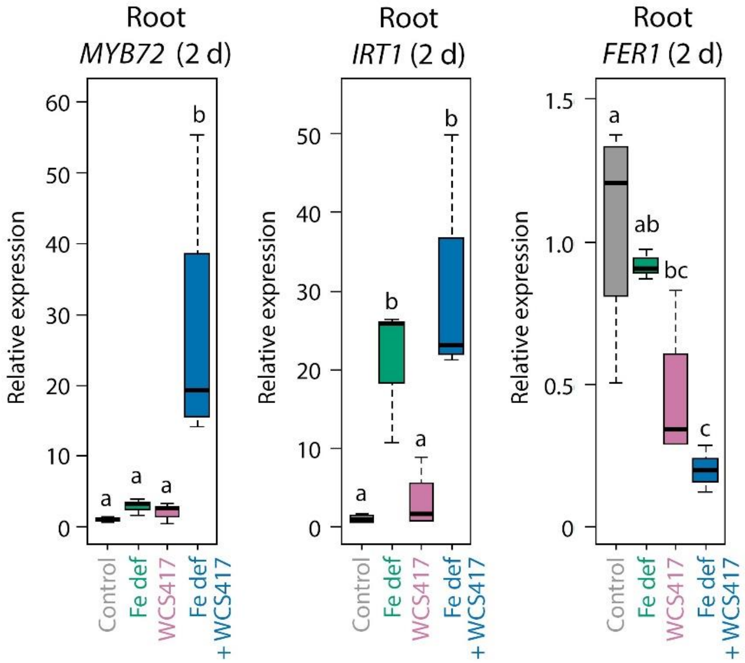

Supplemental Figure S1. ISR and Fe deficiency marker gene expression in response to Fe starvation and WCS417 in roots of plants grown at high density for Fe content measurement. qRT-PCR analysis of *MYB72*, *IRT*, and *FER1* gene expression in roots, 2 days after transfer of 12-day-old Col-0 plants grown in high density on Fe-sufficient plants to either fresh Fe-sufficient plates (Control), to Fe-deficient plates (Fe-def), to fresh Fe-sufficient plates followed by inoculation of the roots with WCS417 (WCS417), or to Fe-deficient plates followed by inoculation with WCS417 (Fe def+WCS417). Gene expression levels were normalized to that of the constitutively expressed reference gene *PP2AA3* (At1g13320). Plotted are fold-changes in gene expression levels relative to that of the average of the control treatment. Error bars represent standard errors of the mean. Different letters indicate statistically significant differences between treatments (One-way ANOVA followed by Tukey's test; $P<0.05$; n=3-4).
